# Supplementary material for: HIVprotI: an integrated web based platform for prediction and design of HIV proteins inhibitors
Source: J Cheminform. 2018 Mar 9;10:12. doi: 10.1186/s13321-018-0266-y (PMC5845081; doi:10.1186/s13321-018-0266-y)
Supplement: Supplementary file 2 — Additional file 2. Source code of HIVProtI web server. [file 13321_2018_266_MOESM2_ESM.zip › HIVprotI_Source-code/hivprotI/weka-3-6-6/doc/weka/classifiers/trees/lmt/LogisticBase.html]

LogisticBase


---


|  |  |  |
| --- | --- | --- |
| **Overview** \| **Package** \| **Class** \| **Tree** \| **Deprecated** \| **Index** \| **Help** \| **Weka's home** | |  |
| **PREV CLASS**   **NEXT CLASS** | **FRAMES**    **NO FRAMES**     **All Classes** |  |
| SUMMARY: NESTED \| FIELD \| CONSTR \| METHOD | DETAIL: FIELD \| CONSTR \| METHOD |  |


---


## weka.classifiers.trees.lmt Class LogisticBase

```
java.lang.Object
  weka.classifiers.Classifier
      weka.classifiers.trees.lmt.LogisticBase
```

**All Implemented Interfaces:**: java.io.Serializable, java.lang.Cloneable, CapabilitiesHandler, OptionHandler, RevisionHandler, WeightedInstancesHandler

**Direct Known Subclasses:**: FTtree, LMTNode

---

``` public class LogisticBase extends Classifier implements WeightedInstancesHandler ```

Base/helper class for building logistic regression models with the LogitBoost algorithm.
Used for building logistic model trees (weka.classifiers.trees.lmt.LMT)
and standalone logistic regression (weka.classifiers.functions.SimpleLogistic).
Valid options are:

```
 -D
  If set, classifier is run in debug mode and
  may output additional info to the console
```

**Version:**
:   $Revision: 1.9 $

**Author:**
:   Niels Landwehr, Marc Sumner

**See Also:**: Serialized Form

---

**Constructor Summary**

`LogisticBase()`
  
          Constructor that creates LogisticBase object with standard options.

`LogisticBase(int numBoostingIterations,
boolean useCrossValidation,
boolean errorOnProbabilities)`
  
          Constructor to create LogisticBase object.


| **Method Summary** | |
| --- | --- |
| `void` | `buildClassifier(Instances data)`             Builds the logistic regression model usiing LogitBoost. |
| `void` | `cleanup()`             Cleanup in order to save memory. |
| `double[]` | `distributionForInstance(Instance instance)`             Returns class probabilities for an instance. |
| `int` | `getMaxIterations()`             Returns the maxIterations parameter. |
| `int` | `getNumRegressions()`             The number of LogitBoost iterations performed (= the number of simple regression functions fit). |
| `java.lang.String` | `getRevision()`             Returns the revision string. |
| `boolean` | `getUseAIC()`             Get the value of useAIC. |
| `int[][]` | `getUsedAttributes()`             Returns an array of the indices of the attributes used in the logistic model. |
| `double` | `getWeightTrimBeta()`             Get the value of weightTrimBeta. |
| `double` | `percentAttributesUsed()`             Returns the fraction of all attributes in the data that are used in the logistic model (in percent). |
| `void` | `setHeuristicStop(int heuristicStop)`             Sets the option "heuristicStop". |
| `void` | `setMaxIterations(int maxIterations)`             Sets the parameter "maxIterations". |
| `void` | `setUseAIC(boolean c)`             Set the value of useAIC. |
| `void` | `setWeightTrimBeta(double w)`             Sets the option "weightTrimBeta". |
| `java.lang.String` | `toString()`             Returns a description of the logistic model (i.e., attributes and coefficients). |

**Methods inherited from class weka.classifiers.Classifier**

`classifyInstance, debugTipText, forName, getCapabilities, getDebug, getOptions, listOptions, makeCopies, makeCopy, setDebug, setOptions`

**Methods inherited from class java.lang.Object**

`equals, getClass, hashCode, notify, notifyAll, wait, wait, wait`

**Constructor Detail**

### LogisticBase

```
public LogisticBase()
```

:   Constructor that creates LogisticBase object with standard options.

---


### LogisticBase

```
public LogisticBase(int numBoostingIterations,
                    boolean useCrossValidation,
                    boolean errorOnProbabilities)
```

:   Constructor to create LogisticBase object.

    **Parameters:**: `numBoostingIterations` - fixed number of iterations for LogitBoost (if negative, use cross-validation or stopping criterion on the training data).: `useCrossValidation` - cross-validate number of LogitBoost iterations (if false, use stopping criterion on the training data).: `errorOnProbabilities` - if true, use error on probabilities instead of misclassification for stopping criterion of LogitBoost


**Method Detail**

### buildClassifier

```
public void buildClassifier(Instances data)
                     throws java.lang.Exception
```

:   Builds the logistic regression model usiing LogitBoost.

    :   **Specified by:**: `buildClassifier` in class `Classifier`
    :   **Parameters:**: `data` - the training data **Throws:**: `java.lang.Exception` - if something goes wrong

---


### getUsedAttributes

```
public int[][] getUsedAttributes()
```

:   Returns an array of the indices of the attributes used in the logistic model.
    The first dimension is the class, the second dimension holds a list of attribute indices.
    Attribute indices start at zero.

    :   **Returns:**: the array of attribute indices

---


### getNumRegressions

```
public int getNumRegressions()
```

:   The number of LogitBoost iterations performed (= the number of simple
    regression functions fit).

    :   **Returns:**: the number of LogitBoost iterations performed

---


### getWeightTrimBeta

```
public double getWeightTrimBeta()
```

:   Get the value of weightTrimBeta.

    :   **Returns:**: Value of weightTrimBeta.

---


### getUseAIC

```
public boolean getUseAIC()
```

:   Get the value of useAIC.

    :   **Returns:**: Value of useAIC.

---


### setMaxIterations

```
public void setMaxIterations(int maxIterations)
```

:   Sets the parameter "maxIterations".

    :   **Parameters:**: `maxIterations` - the maximum iterations

---


### setHeuristicStop

```
public void setHeuristicStop(int heuristicStop)
```

:   Sets the option "heuristicStop".

    :   **Parameters:**: `heuristicStop` - the heuristic stop to use

---


### setWeightTrimBeta

```
public void setWeightTrimBeta(double w)
```

:   Sets the option "weightTrimBeta".

---


### setUseAIC

```
public void setUseAIC(boolean c)
```

:   Set the value of useAIC.

    :   **Parameters:**: `c` - Value to assign to useAIC.

---


### getMaxIterations

```
public int getMaxIterations()
```

:   Returns the maxIterations parameter.

    :   **Returns:**: the maximum iteration

---


### percentAttributesUsed

```
public double percentAttributesUsed()
```

:   Returns the fraction of all attributes in the data that are used in the
    logistic model (in percent).
    An attribute is used in the model if it is used in any of the models for
    the different classes.

    :   **Returns:**: the fraction of all attributes that are used

---


### toString

```
public java.lang.String toString()
```

:   Returns a description of the logistic model (i.e., attributes and
    coefficients).

    :   **Overrides:**: `toString` in class `java.lang.Object`
    :   **Returns:**: the description of the model

---


### distributionForInstance

```
public double[] distributionForInstance(Instance instance)
                                 throws java.lang.Exception
```

:   Returns class probabilities for an instance.

    :   **Overrides:**: `distributionForInstance` in class `Classifier`
    :   **Parameters:**: `instance` - the instance to compute the distribution for **Returns:**: the class probabilities **Throws:**: `java.lang.Exception` - if distribution can't be computed successfully

---


### cleanup

```
public void cleanup()
```

:   Cleanup in order to save memory.

---


### getRevision

```
public java.lang.String getRevision()
```

:   Returns the revision string.

    :   **Specified by:**: `getRevision` in interface `RevisionHandler` **Overrides:**: `getRevision` in class `Classifier`
    :   **Returns:**: the revision


---


|  |  |  |
| --- | --- | --- |
| **Overview** \| **Package** \| **Class** \| **Tree** \| **Deprecated** \| **Index** \| **Help** \| **Weka's home** | |  |
| **PREV CLASS**   **NEXT CLASS** | **FRAMES**    **NO FRAMES**     **All Classes** |  |
| SUMMARY: NESTED \| FIELD \| CONSTR \| METHOD | DETAIL: FIELD \| CONSTR \| METHOD |  |


---
